# Intersection of AHR and Wnt Signaling in Development, Health, and Disease

**DOI:** 10.3390/ijms151017852

**Published:** 2014-10-03

**Authors:** Andrew J. Schneider, Amanda M. Branam, Richard E. Peterson

**Affiliations:** School of Pharmacy and Molecular and Environmental Toxicology Center University of Wisconsin, Madison, WI 53705, USA; E-Mails: ajschnei@wisc.edu (A.J.S.); ambranam@wisc.edu (A.M.B.)

**Keywords:** development, disease, toxicity, AHR (aryl hydrocarbon receptor), TCDD (2,3,7,8-tetrachlorodibenzo-*p*-dioxin), Wnt (wingless-related MMTV integration site), β-catenin (CTNNB1), prostate

## Abstract

The AHR (aryl hydrocarbon receptor) and Wnt (wingless-related MMTV integration site) signaling pathways have been conserved throughout evolution. Appropriately regulated signaling through each pathway is necessary for normal development and health, while dysregulation can lead to developmental defects and disease. Though both pathways have been vigorously studied, there is relatively little research exploring the possibility of crosstalk between these pathways. In this review, we provide a brief background on (1) the roles of both AHR and Wnt signaling in development and disease, and (2) the molecular mechanisms that characterize activation of each pathway. We also discuss the need for careful and complete experimental evaluation of each pathway and describe existing research that explores the intersection of AHR and Wnt signaling. Lastly, to illustrate in detail the intersection of AHR and Wnt signaling, we summarize our recent findings which show that 2,3,7,8-tetrachlorodibenzo-*p*-dioxin (TCDD)-induced disruption of Wnt signaling impairs fetal prostate development.

## 1. Introduction

Homologues of the aryl hydrocarbon receptor (*Ahr*) and wingless-related MMTV integration site (*Wnt*) genes are found in animals as diverse as worms, flies, fish, and humans, which not only hints at their importance throughout evolution, but also provides multiple model organisms in which they can be studied [1,2,3]. Extensive research has revealed important roles in development and health for both AHR and Wnt, and has detailed the biochemical mechanisms by which they affect biological outcomes. However, comparatively little research has explored the possibility that part of the normal function of AHR and Wnt might be to regulate each other. Evidence exists of such crosstalk between the AHR and Wnt homologues in invertebrates [4,5,6], but our review focuses strictly on the intersection of AHR and Wnt signaling in vertebrates, where each pathway is activated by ligand-receptor binding events and can trigger substantial change to the transcriptome. Signaling through each pathway must be precisely regulated because both insufficient and excessive activation can lead to developmental defects and/or disease.

## 2. AHR and Wnt Signaling in Development, Health, and Disease

Wnt signaling is necessary during embryonic development. Knockout of Wnt signaling ligands can cause a multitude of birth defects and even embryonic lethality, while excessive Wnt signaling can also cause developmental defects [7,8,9]. Knockout of AHR is considerably less detrimental during embryonic development. Embryos are viable, but some developmental irregularities and long-term health effects arise in AHR knockout mice, rats, and fish [10,11,12,13,14]. By comparison, over-activation of AHR leads to embryonic lethality or severe developmental defects in multiple tissues, including kidney, palate, teeth, prostate, lungs, heart, and vasculature [15,16,17,18,19,20,21,22].

Wnt signaling is frequently found to be over-active in many types of cancer [23]. AHR signaling has been shown to have a functional role in tumor development, as well, and multiple AHR agonists are classified as carcinogens [24,25,26]. Paradoxically, in some model systems, AHR is a tumor suppressor and activation of AHR protects against tumor development [26,27,28,29,30,31,32]. These apparently contrasting observations can be reconciled if they are considered to be part of a continuum. It seems that a certain level of AHR signaling is necessary for health, but excessive or insufficient AHR signaling can lead to tumor development. Future studies designed to understand if and how AHR and Wnt signaling pathways intersect could shed light on mechanisms of tumor initiation, promotion, and progression while simultaneously providing potential targets for treatment.

Due to the importance of Wnt signaling and the teratogenic potential of AHR signaling during embryonic development, it is reasonable to hypothesize that over-activation of AHR signaling alters Wnt signaling. There is a growing collection of evidence that supports this hypothesis, which is discussed below.

## 3. AHR Signaling

AHR is expressed in many tissues during embryonic development and in adulthood [33,34,35,36,37], and is activated by ligand binding. AHR is a promiscuous receptor capable of binding hundreds of different chemical compounds (for detailed discussion see: [13,14,38]). Many AHR ligands are anthropogenic xenobiotics, including 2,3,7,8-tetrachlorodibenzo-*p*-dioxin (TCDD). TCDD is a common environmental contaminant and considered the prototypical AHR agonist due to its high binding affinity for AHR [14,39,40]. TCDD can hyper activate AHR signaling and therefore, exposure to TCDD during embryogenesis can be teratogenic [15,16,17,18,19,20,21,22].

In the absence of ligand, AHR is complexed with multiple chaperone proteins (HSP90, AIP, and p23) in the cytoplasm [13,38,39]. Ligand binding causes AHR to translocate to the nucleus, shed the chaperone proteins, and bind with aryl hydrocarbon receptor nuclear translocator (ARNT). The AHR-ARNT heterodimer binds to dioxin response elements (DREs) in the promoter region of downstream target genes and directly upregulates their expression [38]. Activated AHR is also capable of downregulating expression, and multiple studies have reported a larger number of genes being downregulated rather than upregulated following AHR activation [41,42,43,44,45,46,47]. However, it is less clear whether transcriptional downregulation is the result of AHR binding directly to the promoter, or if it is an indirect effect due to (1) AHR-induced upregulation of transcriptional inhibitors or (2) AHR-induced degradation of other transcriptional activators (discussed below). Though there are some standard target genes—cytochrome P450 (Cyp)1a1, *Cyp1a2*, and *Cyp1b1*—that indicate activation of AHR signaling, the precise transcriptional response can vary depending on the species [48,49,50,51], tissue or cell type [52,53], activating ligand [41,42,48,52,54], and AHR allele [48,55,56,57].

In addition to direct transcriptional regulation, activated AHR also has E3 ubiquitin ligase activity. Once activated, AHR has been shown to associate with androgen receptor (AR), estrogen receptor alpha (ESR1), or β-catenin (CTNNB1) in a protein complex that results in ubiquitination and, ultimately, degradation of these proteins [28,58]. Thus, activated AHR can downregulate expression of AR, ESR1, and CTNNB1 without affecting the transcriptional activity of these genes. Furthermore, since all three of these genes regulate transcription, activated AHR can, theoretically, indirectly alter transcription of genes that do not necessarily contain a DRE in their promoter region.

## 4. Wnt Signaling

Like AHR signaling, Wnt signaling is activated by ligand-receptor binding, but rather than chemical ligands, Wnt signaling is activated by proteins from the Wnt and R-spondin families. Receptors for these ligands include proteins from the frizzled (FZD), low-density lipoprotein receptor protein (LRP), leucine-rich repeat containing G coupled-protein receptor (LGR), and receptor tyrosine kinase-like orphan receptor (ROR) families [59,60,61,62,63]. Wnt signaling is transduced through multiple intracellular signaling cascades that affect diverse cellular responses including gene transcription, proliferation, differentiation, and migration [64,65,66]. Wnt signaling cascades are defined as canonical (signaling through β-catenin) or non-canonical (signal transduction independent of β-catenin). Of the studies we found that examined the intersection of AHR and Wnt signaling, a large majority were focused on the canonical Wnt pathway, and therefore, this review does the same. However, several studies presented evidence that AHR and non-canonical Wnt signaling might also intersect, suggesting future studies should not exclude this possibility.

Canonical Wnt signaling is transmitted via β-catenin (CTNNB1), a multifunctional protein that, in the context of canonical Wnt signaling, serves as a transcriptional co-activator [67,68]. CTNNB1 expression is tightly regulated in the cytoplasm by the destruction complex which actively downregulates CTNNB1 expression when the canonical Wnt signaling cascade is silent. The destruction complex consists of the scaffold proteins adenomatous polyposis coli (APC) and axin (AXIN1 or AXIN2), plus the serine/threonine kinases, glycogen synthase kinase 3 beta (GSK3B) and casein kinase 1 alpha (CSNK1A1). Both GSK3B and CSNK1A1 phosphorylate CTNNB1, priming it for subsequent ubiquitination and proteasomal degradation. For a detailed description of CTNNB1 degradation see: [69,70,71,72]. Activation of the canonical Wnt signaling cascade inactivates the destruction complex, thereby preventing CTNNB1 phosphorylation. The result is intracellular accumulation of stabilized, non-phosphorylated CTNNB1, which translocates to the nucleus and activates transcription of downstream target genes. The list of CTNNB1 target genes is extensive and varies depending on cell type, but often includes *Axin2*, lymphoid enhancer binding factor 1 (*Lef1*), and myelocytomatosis oncogene (*Myc*) [73,74,75,76].

## 5. Assessing AHR and Wnt Signaling Pathways

As discussed above, there are hundreds of potential agonists and antagonists of AHR. This review only includes studies that used well-known AHR agonists and/or demonstrated by AHR knockdown or knockout that the effects being studied were due to AHR activation. However, there are numerous other studies (including [39,77,78,79,80,81]) that have reported altered Wnt signaling after exposure to either (1) a known AHR agonist that also can act independently of AHR, or (2) a potential AHR agonist, but the effects on Wnt signaling induced by these compounds were not confirmed to be mediated through AHR. For example, addition of indole-3-carbinol (I3C) to the culture media of a human prostate cancer cell line downregulated CTNNB1 expression [82]. While I3C can bind to AHR [40,83], several studies have shown that it is a poor agonist that does not necessarily activate AHR signaling [84,85,86,87]. The acid condensation products of I3C, *i.e.* 3,3'-diindolylmethane (DIM), have higher affinity for AHR [40,83], but it is not obvious that I3C could form these condensation products in cell culture. Furthermore, neither AHR expression nor AHR target gene expression was assessed in this study, so it is not clear if I3C-induced downregulation of CTNNB1 was AHR dependent. Another example is indirubin-3'-monoxime, an analog of indirubin and a known AHR agonist [88], can also activate the canonical Wnt signaling pathway, likely by inhibiting GSK3B function [89,90,91]. However, it is not known if this function of indirubin-3'-monoxime is AHR-dependent. Ideally future experiments will clearly define the role of AHR in model systems being exposed to potential AHR agonists.

When analyzing the canonical Wnt signaling pathway, there are at least three aspects that should be considered: (1) activation of the cascade upstream of CTNNB1; (2) CTNNB1 stabilization and nuclear localization; and (3) downstream transcriptional changes induced by CTNNB1. Evaluating only one of these aspects when reporting on activation or alteration of canonical Wnt signaling can be misleading. For example, reporting that there is a change in ligand expression does not confirm that this change is functionally relevant to downstream target gene expression. Similarly, a change in CTNNB1 expression does not guarantee a functional change in target gene transcription. Furthermore, it is not just the amount but also the intracellular location (nucleus) of CTNNB1 expression that is important for canonical Wnt signaling to occur. And lastly, only reporting on transcriptional activity of CTNNB1 target genes can be misleading because there are multiple signaling pathways that can transduce their signal by triggering stabilization of CTNNB1 [68,92,93]. Critically, activation of AHR can alter signal transduction and CTNNB1 stability through these alternate pathways [94,95,96]. Therefore, analyzing activity upstream of CTNNB1, CTNNB1 expression and localization, and activity downstream of CTNNB1 are all important to properly conclude that activation or alteration of the canonical Wnt signaling cascade has occurred. It is important to note that many studies reviewed here do not fully analyze all three aspects and therefore, to some degree, infer the activation or alteration of canonical Wnt signaling without actually confirming it. Despite this caveat, these studies have been included because they begin to provide a description of the intersection of Wnt and AHR signaling.

## 6. Wnt Signaling Effects on AHR Signaling

Activation of the canonical Wnt signaling pathway can upregulate transcription and expression of *Ahr* in multiple cell types. WNT3A, lithium chloride (LiCl, a known GSK3B inhibitor), and CTNNB1 with stabilizing mutations can all activate, or mimic activation of the canonical Wnt signaling cascade by promoting intracellular accumulation and nuclear localization of CTNNB1. When six different cell lines from four different tissue sources [97,98,99,100,101] and primary mouse hepatocytes [99,102] were cultured with any one of these activators, *Ahr* transcription and/or expression was upregulated. Furthermore, AHR expression *in vivo* was linked to canonical Wnt signaling in rodent livers. Within the liver, blood flows from portal veins to central veins creating a porto-central axis [103]. Hepatocytes surrounding the portal veins (periportal zone) express a proteome different from that of hepatocytes surrounding central veins (perivenous zone). This is in part due to canonical Wnt signaling which is active in the perivenous zone, but not the periportal zone [102,103,104,105]. AHR is expressed primarily in the perivenous zone [106,107,108] and transcription of *Ahr* is reduced in mice with hepatocyte-specific CTNNB1 knockout [99,105,108], which suggests that AHR expression is at least partially regulated by canonical Wnt signaling *in vivo*. Additionally, liver tumors overexpressing CTNNB1 also express elevated levels of AHR while liver tumors that do not overexpress CTNNB1 do not show excessive AHR expression [108,109].

Identifying *Ahr* as a CTNNB1 target gene is relevant to the discussion of how Wnt and AHR signaling intersect, but it does not actually demonstrate whether or not Wnt signaling affects AHR signaling. Several studies explored this possibility by looking at reporter gene or target gene (*Cyp1a1 and/or Cyp1b1*) activity. In a mouse hepatoma cell line, TCDD activated expression of an AHR reporter gene (luciferase driven by a promoter containing three DREs) to a significantly greater extent in cells expressing a stabilized CTNNB1 (CTNNB1^S33Y^ mutation) than in control cells expressing wild type CTNNB1 (Table 1) [110]. Thus, CTNNB1 can boost AHR-induced transcription, and can do so by enhancing AHR activity at DREs. Similar results were reported in primary mouse hepatocytes and in a multipotent stem cell-like cell line derived from rat liver (WB-F344 cells). Culture with TCDD plus WNT3A activated *Cyp1a1* transcription in both cell types to a greater degree than culture with TCDD alone (Table 1 and Table 2) [99,100]. WNT3A also enhanced TCDD-induced *Cyp1a1* expression and *Cyp1b1* transcription and expression in WB-F344 cells [100]. Furthermore, knockdown of CTNNB1 in two mouse hepatoma cell lines, as well as *in vivo* knockout of CTNNB1 in mouse hepatocytes, both resulted in a significantly weaker upregulation of *Cyp1a1* transcription after activation of AHR [99].

While the above studies demonstrate that CTNNB1 can enhance AHR signaling, and in fact, plays a role in the endogenous response to xenobiotic exposure, canonical Wnt signaling had no effect on AHR signaling in human prostate cancer cell lines (Table 1) [97]. CYP1A1 expression in TCDD-exposed cells treated with WNT3A was identical to that of TCDD-exposed cells not treated with WNT3A. However, counter-intuitively, TCDD completely repressed CYP1A1 expression in these cells, therefore they might not serve as the best model in which to study Wnt signaling effects on AHR signaling.

**Table 1:** Intersection of AHR and WntSignaling.

Species	Tissue/Cell Type	Activated AHR Alters Wnt Signaling	Wnt Signaling Alters AHR Signaling	Reference
Human	Prostate cancer cell lines	-	No	[97]
Mouse	Hepatoma cell line	-	Yes (Up)	[110]
Mouse	Hepatoma cell lines; Primary hepatocytes; Hepatocyte-specific CTNNB1 knockout mice	No	Yes (Up)	[99] ^g^
Rat	Multipotent stem cell-like cell line isolated from liver	Yes (Down)	Yes (Up)	[100] ^g^
Mouse & Human	Intestinal tissue & tumors from *Ahr ^−^*^/*−*^ & *Apc ^min^*^/*+*^ mice; Human colon cancer cell lines	Yes (Down)	-	[28] ^g^
Rat	Multipotent stem cell-like cell line isolated from liver	Yes (Down)	-	[47] ^g^
Rat	Brain (cortex); Primary cortical neurons; Pheochromocytoma cell line (adrenal gland) ^a^	Yes (Down)	-	[129] ^g^
Human	Placental choriocarcinoma & endometrial adenocarcinoma cell lines	Yes (Down)	-	[130] ^g^
Human	Breast cancer cell line (mammospheres)	Yes (Down) ^b^	-	[131] ^g^
Mouse	Urogenital sinus	Yes (Down)	-	[143] ^g^
Mouse	Urogenital sinus	Yes (Down)	-	[152] ^g^
Zebrafish	Fin (regeneration)	Yes (Up)	-	[44] ^g^
Mouse	Embryonal carcinoma cell line	Yes (Up) ^c^	-	[116] ^g^
Human	Prostate cancer cell line	Yes (Up) ^d^	-	[41]
Mouse	Palate	Yes (Down) ^d^	-	[124]
Mouse	Primary lung fibroblast	Yes ^e^	-	[132] ^g^
Mouse	Urogenital sinus	No ^d,f^	-	[153]
Zebrafish	Swim bladder	Inconclusive	-	[190]

^a^ Differentiated into neuronal cells. ^b^ AHR was mutated to be constitutively active, independent of ligand. ^c^ Knockdown of AHR decreased *Ctnnb1* mRNA abundance. ^d^ *Wnt5a* was the primary gene of interest in these studies. ^e^ There is evidence of both upregulation and downregulation of Wnt signaling in this system. ^f^ TCDD did not alter WNT5A expression, but it was hypothesized that TCDD did alter the balance of Wnt signaling. ^g^ Additional information in Table 2.

**Table 2:** Effects of Activated AHR on Canonical Wnt Signaling.

Direction of Altered Wnt Signaling	Upstream Regulators of Wnt Signaling	CTNNB1 Expression and Nuclear Localization	Downstream CTNNB1 Target Genes	Reference
Down	p-DVL2 (↓) ^a^		*Lef1* (↓)	[100]
	p-DVL3 (↓) ^a^	Expression (↓)	*Axin2* (↓)	
	p-GSK3B (nc) ^a,b^	Nuclear Localization (↓)	*Ccnd1* (↓)	
	p-LRP6 (nc) ^a,b^		Other target genes	
Down	- ^c,d^	Expression (↓) ^d^	CTNNB1 reporter gene (↓)	[28]
Down	*Wnt4* (↓)	-	*Lef1* (↓) *Axin2* (↓) Other potential target genes	[47]
	*Wnt5a* (↓)			
	*Fzd1* (↓)			
	*Fzd4* (↓)			
Down	p-GSK3B (↓) ^a^	Expression (↓)	-	[129]
Down	-	Expression (↓)	-	[130]
Down	-	Expression (↓) ^e^	-	[131]
Down	*Rspo2* (↓)	-	*Lef1* & LEF1 (↓) *Hnf1a* & HNF1A (↓) *Wif1* (↓)	[143]
	*Rspo3* (↓)			
	*Lgr5* (↓)			
	*Lgr4* (nc)			
Down	*Wnt10a* (↑)		LEF1 (↓) *Wif1* (↓) *Ror2* (↓)	[152]
	*Wnt16* (↑)	Expression (↓)		
	*Rspo2* (↓)	Nuclear Localization (↓)		
	all other Wnts (nc)			
Up	*rspo1* (↑)	-	-	[44]
	*lrp6* (nc) ^f^			
Up	-	Expression (↓) ^g^	-	[116]
Up ^h^	*Wnt5a* (↓) *Wnt5b* (↓) *Wnt9a* (↓)	-	*Lef1* (↑)	[132]
			*Axin2* (↑)	
			*Myc* (↑)	
			*Wisp2* (↑)	
No Effect	-	-	*Axin2* (nc)	[99]
			*Lgr5* (nc)	
			CTNNB1 reporter gene (nc)	

^a^ “p” represents the phosphorylated form of the protein. Generally, an increase in phosphorylation for each of these proteins indicates an increase in canonical Wnt signaling. DVL = disheveled. ^b^ nc = no change. ^c^ “-” = not tested. ^d^ AHR downregulates CTNNB1 expression by inducing CTNNB1 ubiquitination and degradation, not by altering the upstream signal. ^e^ AHR was mutated to be constitutively active, independent of ligand. ^f^ RNA abundance was not formally tested after exposure to TCDD, but was not identified as different in a microarray experiment. ^g^ *Ahr* knockdown caused a decrease in *Ctnnb1* mRNA abundance. ^h^ We hypothesize canonical Wnt signaling was upregulated based on increased downstream target gene RNA levels.

## 7. Effects of Activated AHR on Wnt Signaling

### 7.1. Upregulation of Wnt Signaling

One of the first studies to provide strong evidence that AHR signaling could differentially regulate Wnt signaling was performed in zebrafish (Table 1 and Table 2) [44]. The caudal fin of zebrafish regenerates after amputation. However, exposure to TCDD after fin amputation severely impairs the regeneration process. Microarray analysis of mRNA from the regenerating fins of control and TCDD-exposed larval fish revealed that TCDD altered transcription of multiple Wnt signaling regulators, including *rspo1*, which was upregulated. Morpholino-induced knockdown of Rspo1 in TCDD-exposed, fin-amputated larvae permitted normal fin regeneration, thereby demonstrating that overexpression of this single gene was sufficient to inhibit the regenerative process [44]. Rspo1 had previously been identified as an activator of canonical Wnt signaling [111,112,113,114] so it was hypothesized that TCDD inhibited fin regeneration in zebrafish by activating Ahr2, which upregulated Rspo1 expression, which, in turn, induced canonical Wnt signaling [44]. Multiple canonical Wnt signaling target genes were differentially regulated in the same microarray that found *rspo1* to be upregulated, thus providing support for the hypothesis that TCDD indirectly induced canonical Wnt signaling. However, no alternative methods were used to test the transcription or expression of these target genes so as to confirm their upregulation. Instead, larval fin amputees were treated with the GSK3 inhibitor, (2'Z,3'E)-6-bromoindirubin-3'-oxime (BIO), to upregulate canonical Wnt signaling. BIO severely impaired fin regeneration, thereby phenocopying TCDD exposure, and further supporting the hypothesis that active canonical Wnt signaling inhibits fin regeneration [44]. Importantly, downregulation of canonical Wnt signaling also inhibited zebrafish fin regeneration [115]. Thus, it seems that the caudal fin regeneration model is a good example of a tissue where both insufficient and excessive canonical Wnt signaling impairs development. Activation of Ahr2 appears to disrupt the balance of canonical Wnt signaling by promoting over-activation.

We found only one other instance where AHR signaling might upregulate canonical Wnt signaling. A multipotent mouse embryonal carcinoma cell line expressed both AHR and CTNNB1, and was made to differentiate into cardiomyocytes. *Ctnnb1* mRNA levels increase after 10 days of differentiation, but in cells with AHR knocked down, *Ctnnb1* mRNA levels decrease after 10 days of differentiation (Table 1 and Table 2) [116]. Thus, in this cell line, it appears that AHR at least partially controls transcription of *Ctnnb1* during differentiation into cardiomyocytes, and therefore might promote canonical Wnt signaling.

Exposing a human prostate cancer cell line to the AHR agonists TCDD and/or benzo[*a*]pyrene (BaP) rapidly induced WNT5A expression and increased mRNA levels of *FZD1*, *FZD3*, and *LEF1* (Table 1) [41]. WNT5A is typically found to activate a non-canonical Wnt signaling cascade, but has also been shown to activate canonical Wnt signaling [117,118,119]. LEF1 has been identified as a canonical Wnt signaling target gene in the prostate [120,121] so it is possible that WNT5A or some other ligand is activating canonical Wnt signaling in these cells. Several other studies have also reported elevated expression of both WNT5A and LEF1 [122,123,124]. Though no follow-up experiments were performed to elucidate the precise effects on canonical and/or non-canonical Wnt signaling, it seems likely that activated AHR upregulated WNT5A-induced Wnt signaling in these cells. WNT5A expression was also upregulated in the prostates of seven-year-old Rhesus macaques after *in utero* and lactational exposure to TCDD (Table 1) [45]. This increased WNT5A expression was associated with increased fibrosis, and decreased glandularity in the prostate.

### 7.2. Downregulation of Wnt Signaling

Of the studies shown in Table 1, only two addressed both sides of the AHR-Wnt intersection [99,100]. While canonical Wnt signaling was found to enhance AHR signaling *in vivo* in mouse liver and in a mouse hepatoma cell line, activated AHR did not alter canonical Wnt signaling. Specifically, exposure to TCDD did not affect mRNA levels of CTNNB1 target genes *Axin2* and *Lgr5* in the hepatoma cell line, nor did 3-methylcholanthrene (3MC, an AHR agonist) affect mRNA levels of the same two target genes in the liver [99]. These findings were reported as unpublished data. In the multipotent WB-F344 cell line, canonical Wnt signaling similarly enhanced AHR signaling. However, TCDD-induced activation of AHR signaling decreased canonical Wnt signaling by downregulating expression of CTNNB1 and transcription of multiple downstream CTNNB1 target genes. This downregulation was AHR-dependent but likely was not due to the E3 ubiquitin ligase activity of AHR [100].

Activated AHR has also been shown to downregulate canonical Wnt signaling in mouse intestines, where it appears to have a perpetual and necessary role as an inhibitor of canonical Wnt signaling (Table 1 and Table 2) [28]. C57BL/6 mice injected with the AHR agonists 3MC, indole-3-acetic acid (IAA), or indole-3-carbinol (I3C) all had decreased CTNNB1 expression in the cecum compared to control mice. CTNNB1 expression in *Ahr* knockout mice (*Ahr^−^*^/*−*^) was unaffected after 3MC injection, demonstrating that CTNNB1 downregulation is AHR dependent. Additionally, untreated *Ahr^−^*^/*−*^ mice overexpress CTNNB1 and the CTNNB1 target gene, *Myc*, in the ileum, cecum, and colon compared to *Ahr* wild type (*Ahr^+^*^/*+*^) mice. Overactive canonical Wnt signaling can lead to tumor development in the intestines [23,125], which could explain why *Ahr^−^*^/*−*^ mice spontaneously develop tumors in the cecum. Thus, AHR has a natural function as a tumor suppressor that is capable of downregulating CTNNB1 expression. *Apc-min* (multiple intestinal neoplasia) mice have a mutated *Apc* gene which leads to a dysfunctional CTNNB1 destruction complex, overexpression of CTNNB1, and development of intestinal tumors [28,126,127,128]. Compound mutant mice with one or both *Ahr* alleles knocked out (*Apc^min^*^/*+*^; *Ahr^+^*^/*−*^ and *Apc^min^*^/*+*^; *Ahr^−^*^/*−*^, respectively) overexpress CTNNB1 and develop cecal tumors at a younger age than *Apc^min^*^/*+*^; *Ahr^+^*^/*+*^ mice, providing additional evidence that AHR acts as a tumor suppressor and downregulates CTNNB1 expression in the intestines. Furthermore, I3C or 3,3'-diindolylmethane (DIM, an AHR agonist) delayed time to tumor development and reduced tumor multiplicity when added to the diet of *Apc^min^*^/*+*^; *Ahr^+^*^/*+*^ or *Apc^min^*^/*+*^; *Ahr^+^*^/*−*^ mice, but these AHR agonists had no effect on tumor development in *Apc^min^*^/*+*^; *Ahr^−^*^/*−*^ mice. Activated AHR also downregulated CTNNB1 expression in multiple human colon cancer cell lines. These cell lines were used to demonstrate that activated AHR downregulated CTNNB1 by binding to and promoting ubiquitination of CTNNB1. The ubiquitinated CTNNB1 subsequently underwent proteasomal degradation. Interestingly, immunohistological analysis of human cecal tumors revealed decreased AHR expression and increased CTNNB1 expression in 12/12 samples tested [28].

Several studies have identified detrimental biological outcomes caused by exposure to TCDD and have linked those outcomes to downregulation of canonical Wnt signaling. For example, exposure to TCDD induced neuronal apoptosis *in vivo* in the rat cortex, in primary cortical neurons isolated from rat, and in the rat PC12 cell line [129]. TCDD exposure also decreased GSK3B phosphorylation (thus increasing the pool of active GSK3B) and decreased CTNNB1 expression (Table 1) [129]. Treatment with LiCl countered the effects of TCDD by increasing GSK3B phosphorylation and CTNNB1 expression, and reducing apoptosis. Thus, neuronal apoptosis was linked to downregulation of canonical Wnt signaling. However, it was not determined whether or not Wnt ligand expression upstream of CTNNB1 was altered or if downstream CTNNB1 target gene expression was altered (Table 2).

TCDD also was found to disrupt attachment of spheroids generated from human placental choriocarcinoma cell lines to adherent human endometrial adenocarcinoma cell lines in a culture model designed to mimic embryo implantation [130]. TCDD downregulated CTNNB1 expression in the spheroids, but WNT3A or LiCl reversed the inhibitory effects of TCDD by increasing both CTNNB1 expression and the percentage of spheroids that attached to endometrial cells (Table 1). Thus, successful spheroid attachment was associated with active canonical Wnt signaling. However, downstream target gene expression was not analyzed (Table 2). CTNNB1, in addition to regulating transcription, has a second Wnt-independent function in maintaining cell-cell adhesion as a component of adherens junctions [67]. Testing downstream target gene expression would help elucidate if CTNNB1 was promoting spheroid attachment through transcriptional regulation or was directly participating in cell-cell adhesion.

Expression of a mutated AHR designed to be constitutively active, even in the absence of ligand, downregulated CTNNB1 expression in mammospheres generated from a human breast cancer cell line (Table 1) [131]. This was associated with a decreased capacity for the mammospheres to self-renew. Neither upstream activation, nor downstream target gene expression was tested to confirm altered canonical Wnt signaling (Table 2).

There is also some evidence that activated AHR can downregulate *Wnt5a*. Both WNT5A and LEF1 expression were reported to be downregulated in developing mouse palate after exposure to TCDD (Table 1) [124]. While the paper provides a nice description of the physical changes that occur in the developing palate after TCDD exposure, the conclusions that WNT5A and LEF1 are downregulated are somewhat dubious because they were based on a subjective reading of a Western blot that was not quantitated. However, microarray analysis of primary mouse lung fibroblasts cultured with TCDD or 2-(1'H-indolo-3'-carbonyl)-thiazole-4-carboxylic acid methyl ester (ITE; an AHR agonist) revealed decreased *Wnt5a* mRNA levels (Table 2) [132]. Interestingly, a cursory check of the gene list showed that *Wnt5b* and *Wnt9a* were also downregulated, while multiple genes commonly identified as canonical Wnt signaling target genes, including *Lef1*, *Axin2*, *Myc*, and Wnt1 inducible signaling pathway 2 (*Wisp2*) [133] were all upregulated, suggesting that activated AHR did alter Wnt signaling (Table 2). The biological significance of these findings is not known. Microarray analysis of the multipotent stem cell-like cell line, WB-F344, after exposure to 3,3',4,4',5-pentachlorobiphenyl (PCB126, an AHR agonist) also found *Wnt5a* to be downregulated, along with *Wnt4*, *Fzd1*, and *Fzd4* (Table 1 and Table 2) [47]. Numerous other genes linked to Wnt signaling were also downregulated including *Lef1* and *Axin2*. While this provides strong evidence that activated AHR downregulates Wnt signaling, it is not clear which Wnt cascade or cascades are being dysregulated, or what the biological significance of this finding is.

## 8. AHR and Wnt Signaling in Mouse Prostate Development

**Figure 1:**
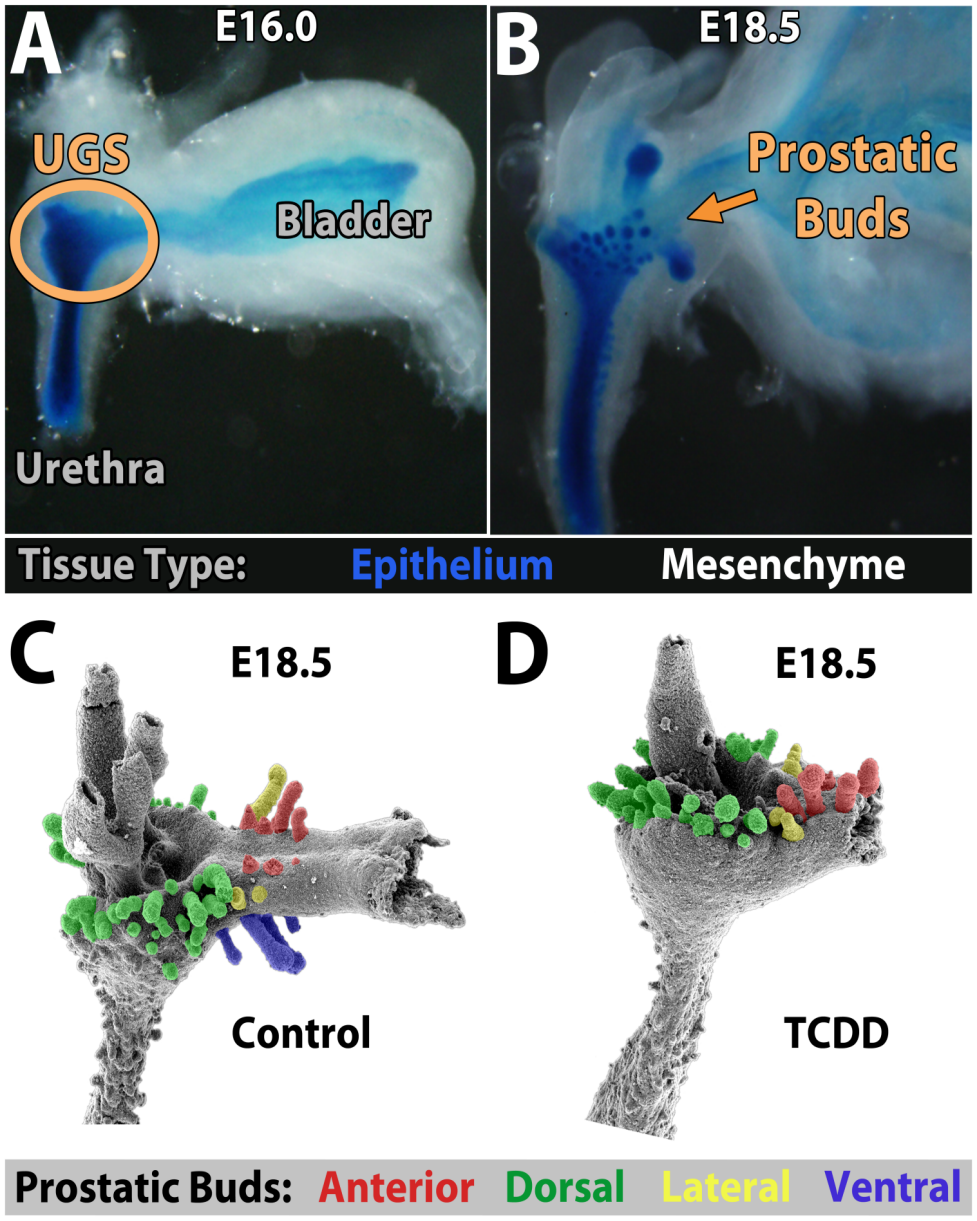
*In vivo* prostatic bud development in male C57BL/6J mouse embryos. (**A**) The urogenital sinus (UGS) is visible between the bladder and urethra. Epithelium is stained blue; mesenchyme is white. At E16.0, prostatic budding has not yet initiated. (**B**) By E18.5, all prostatic buds have formed. (**C**,**D**) Dams were dosed on E15.5 with corn oil vehicle (5 mL/kg, po; (**C**)) or 2,3,7,8-tetrachlorodibenzo-*p*-dioxin (TCDD) (5 μg/kg, po; (**D**)). UGSs were harvested at E18.5, the mesenchyme was removed, and the epithelium was imaged by scanning electron microscopy. Buds are pseudo-colored based on which lobe of the prostate they will form. Images in (**C**) and (**D**) were adapted from [160] with permission from Oxford University Press, copyright 2008.

The adult mouse prostate consists of four pairs of bilateral lobes—the anterior, dorsal, lateral, and ventral lobes. Each develops via a highly orchestrated budding process from the embryonic urogenital sinus (UGS), which is situated between the bladder and pelvic urethra (Figure 1A). The UGS is comprised of a thick layer of epithelium surrounded by mesenchyme that can be divided into multiple subcompartments [134]. As a male secondary sex organ, prostate development is initiated and regulated by androgen signaling [135,136]. Activation of androgen signaling in the UGS mesenchyme (UGM) generates a wave of paracrine signaling that instructs the UGS epithelium (UGE) to start budding (Figure 1A,B) [136,137]. The timing of prostatic budding initiation is not uniform throughout the UGS. It occurs first in the anterior and dorsal regions starting around embryonic day (E) 16.5, and last in the lateral and ventral regions beginning around E17.5 [138]. By E18.5, all prostatic buds have formed (Figure 1B,C). Mouse prostate development is reviewed in more detail elsewhere [137,139,140,141].

**Figure 2:**
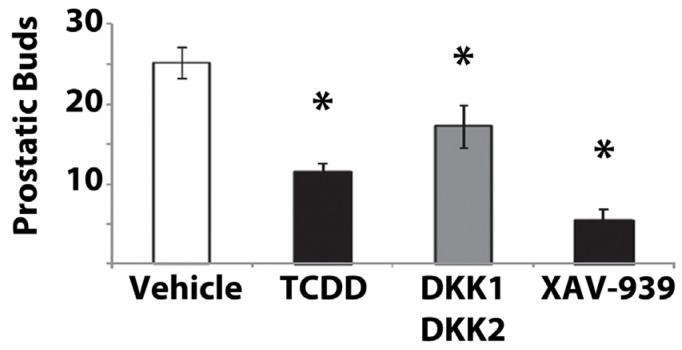
Prostatic bud development *in vitro*. UGSs were harvested from male C57BL/6J mouse embryos at E14.5 and grown in culture containing 5α-dihydrotestosterone (DHT) plus vehicle, TCDD (1 nM), or canonical Wnt inhibitors dickkopf1 (DKK1) and DKK2 (500 ng/mL each) or XAV-939 (10 μM) for four days. All prostatic buds were counted, and an asterisk indicates a significant difference compared to controls (*p* ≤ 0.05). Adapted from [143] with permission from Oxford University Press, copyright 2013.

Canonical Wnt signaling is also essential for prostatic bud formation. UGSs cultured *in vitro* with 5α-dihydrotestosterone (DHT; required to induce budding) plus XAV-939, a chemical inhibitor of canonical Wnt signaling [142], had greater than a 70% decrease in total bud number compared to control UGSs cultured with DHT alone (Figure 2) [143]. Transcript levels of two downstream target genes, *Lef1* and *Lgr5*, were decreased in the XAV-939-exposed UGSs, confirming that XAV-939 downregulated canonical Wnt signaling [143]. UGSs cultured with an excess of the endogenous canonical Wnt signaling inhibitors, dickkopf1 (DKK1) and DKK2 [101], also developed fewer prostatic buds than controls (Figure 2) [143]. Furthermore, three different strategies used to knockout CTNNB1 in the UGS of genetically engineered mice all resulted in impaired prostatic bud formation [60,66,144]. Collectively, these data demonstrate that canonical Wnt signaling is required for prostatic budding.

Interestingly, there are abundant data demonstrating crosstalk between androgen signaling and canonical Wnt signaling, including a physical interaction between androgen receptor and CTNNB1, and transcriptional regulation of one pathway by the other [145,146,147,148]. Thus, altering one pathway could affect the function of the other. However, much of these data were gathered *in vitro* in prostate cancer cell lines. The extent to which these pathways crosstalk *in vivo* in the UGM and/or UGE is not well defined, and, therefore, it is not known if these pathways function independently or in tandem to orchestrate prostatic budding initiation and elongation.

AHR signaling, on the other hand, is not required for prostatic bud formation. Male C57BL/6J mice carrying knockout mutations in both copies of the *Ahr* gene retain normal bud formation and develop functional prostates [138,149]. However, over-activation of AHR signaling during *in utero* development can inhibit prostatic budding. A single dose of TCDD given to pregnant C57BL/6J mice (5 µg/kg dam, po) within the E13.5 to E15.5 developmental window, which is prior to the initiation of prostatic budding, reduces the number of dorsal and lateral buds and completely prevents ventral bud formation (Figure 1D) [138]. AHR knockout mouse fetuses similarly exposed to TCDD undergo normal prostatic budding, confirming that the teratogenic effects of TCDD are AHR dependent. Furthermore, tissue recombination experiments revealed that TCDD-induced activation of AHR signaling in the UGS mesenchyme, but not the UGS epithelium, impaired prostatic budding [150].

While the primary site of action for both androgen signaling and AHR signaling with regards to prostate development is the UGM, activation of AHR signaling in the UGM does not directly impair androgen signaling [151]. Therefore, we searched for other paracrine signaling pathways that were dysregulated by TCDD exposure. Below we discuss our recent findings that TCDD-induced activation of AHR signaling in the UGM downregulates canonical Wnt signaling in the UGE, leading to inhibition of ventral prostatic budding, which manifests as ventral prostate agenesis [143,152].

## 9. Activated AHR Inhibits Canonical Wnt Signaling During Prostatic Budding

### 9.1. In Vitro

*In vitro* TCDD exposure during UGS organ culture mimics *in utero* TCDD exposure by inhibiting prostatic budding. At E14.5 (prior to initiation of prostatic budding), male UGSs were placed in culture containing DHT and either vehicle or TCDD. After four days, prostatic budding was visible in all UGSs, but those exposed to TCDD developed about 50% fewer buds than controls (Figure 2) [143,153]. To determine if TCDD was altering Wnt signaling, mRNA and protein abundance of confirmed canonical Wnt signaling target genes were measured: *Lef1*, *Lgr5*, hepatocyte nuclear factor 1a (*Hnf1a*; aka *Tcf1*), and Wnt inhibitory factor 1 (*Wif1*) [74,75,120,121,144,154,155,156,157,158,159]. TCDD decreased mRNA levels of all four target genes, and, in basal epithelial cells specifically, TCDD reduced expression of LEF1 and HNF1A (Table 1 and Table 2) [143]. We focused on basal epithelial cells because those cells receive and respond to mesenchymally derived paracrine signals and ultimately initiate prostatic budding. Altogether, activation of AHR signaling by TCDD downregulates canonical Wnt signaling and inhibits prostatic bud formation in UGSs cultured *in vitro*.

### 9.2. In Vivo

To demonstrate activation of AHR signaling *in vivo*, transcription of AHR target genes was assessed in male UGSs exposed to TCDD *in utero*. TCDD increased transcription of *Cyp1a1*, *Cyp1b1*, and an AHR reporter gene in the UGE and/or UGM [160]. Because TCDD completely blocked prostatic budding in the ventral UGS, this was the primary region of interest. AHR signaling was not detectably stronger in the ventral region compared to the rest of the UGS [160].

**Figure 3:**
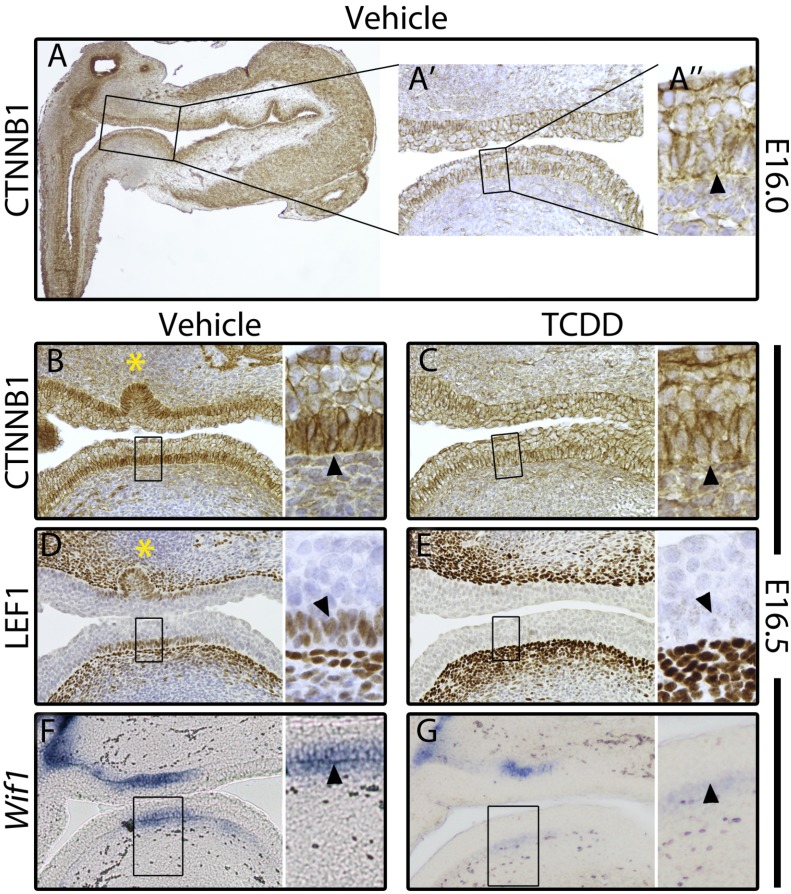
TCDD-induced activation of AHR signaling prevented activation of canonical Wnt signaling in the ventral UGE of male C57BL/6J mouse embryos. Dams were dosed on E15.5 with corn oil vehicle (5 mL/kg, po) or TCDD (5 μg/kg, po). Images are representative sagittal sections from vehicle-exposed (**A**,**B**,**D**,**F**) or TCDD-exposed (**C**,**E**,**G**) UGSs stained by IHC (**A**–**E**, brown stain) or ISH (**F**–**G**, purple stain). (**A**) At E16.0 in both control and TCDD-exposed UGSs, CTNNB1 expression was restricted to cell membranes in the UGE. (**B**) By E16.5 in control UGSs, CTNNB1 had accumulated in the cytoplasm and translocated into the nucleus of ventral basal epithelial cells. (**D**,**F**) CTNNB1 target genes *Lef1* (**D**) and *Wif1* (**F**) were also expressed in the ventral basal epithelial cells of E16.5 control UGSs, demonstrating functionally active CTNNB1 signaling. (**C**) At E16.5 in TCDD-exposed UGSs, CTNNB1 did not accumulate in the cytoplasm, and was not detected in the nucleus of ventral basal epithelial cells. (**E**,**G**) Neither *Lef1* nor *Wif1* was expressed in ventral basal epithelial cells, confirming there is no active CTNNB1 signaling in the ventral epithelium of TCDD-exposed UGSs. Arrowheads point to basal epithelial cells; yellow asterisks mark prostatic buds. Adapted from [152] with permission from Oxford University Press, copyright 2014.

Canonical Wnt signaling was also evaluated in UGSs of male mouse fetuses exposed to vehicle or TCDD *in utero*. As discussed above, activation of canonical Wnt signaling—which is characterized by stabilization, cytoplasmic accumulation, and nuclear localization of CTNNB1—is a process that must occur in the UGS to initiate prostatic budding [66]. Therefore, we stained sagittal sections of UGSs using immunohistochemistry (IHC) to visualize CTNNB1 expression at developmental time points before ventral prostatic buds were present [152]. At E16.0, in both control and TCDD-exposed UGSs, the ventral UGE had no detectable cytoplasmic or nuclear CTNNB1 (Figure 3A). However, by E16.5 in control UGSs, ventral basal epithelial cells showed increased CTNNB1 expression in the cytoplasm and nucleus (Figure 3B). This indicates that the canonical Wnt signaling pathway is first activated in the ventral basal epithelium around E16.5 during normal development. By comparison, CTNNB1 expression in ventral basal epithelial cells of E16.5 TCDD-exposed UGSs resembled that of E16.0 UGSs with little to no detectable CTNNB1 in the cytoplasm or nucleus (Figure 3C). These data show that during normal development, the canonical Wnt signaling cascade is initially activated in basal epithelial cells of the ventral UGS around E16.5, just prior to the initiation of ventral budding, and that *in utero* exposure to TCDD blocks this activation (Table 1 and Table 2).

Though nuclear CTNNB1 expression indicates that the canonical Wnt signaling pathway has been activated, concomitant expression changes of downstream CTNNB1 target genes must also be demonstrated to verify that the pathway is functionally active. Therefore, we assessed expression of the CTNNB1 target gene, LEF1 [74,75,120,121]. In control UGSs, LEF1 was absent in the ventral UGE at E16.0, but by E16.5 most basal epithelial cells in the ventral UGS expressed LEF1, thus illustrating that LEF1 expression temporally coincides with nuclear localization of CTNNB1 and the start of ventral bud formation (Figure 3D) [152]. In TCDD-exposed UGSs, LEF1 expression was not detected in the ventral basal epithelium at either E16.0 or E16.5, which is consistent with the previously described lack of CTNNB1 nuclear localization (Figure 3E and Table 2).

In the UGS, *Wif1* is expressed in both the mesenchyme and epithelium but the transcriptional regulation of this gene is different in each tissue compartment [144,161]. In the epithelium (but not mesenchyme), *Wif1* is upregulated by, and therefore a downstream target gene of, CTNNB1 signaling [144]. As such, *Wif1* transcript was detected by *in situ* hybridization (ISH) in the UGE of E16.5 control UGSs, but was greatly reduced in the ventral epithelium of E16.5 TCDD-exposed UGSs (Figure 3F,G) [152]. The TCDD-induced downregulation of *Wif1*, like LEF1, demonstrates that activated AHR inhibited CTNNB1 signaling in the ventral UGS (Table 2). It also provides additional evidence that activated AHR can alter Wnt signaling because WIF1 is an extracellular antagonist of Wnt signaling that binds to Wnt ligands and prevents them from interacting with their cognate receptors [162,163,164]. Presumably, CTNNB1 upregulates WIF1 in the UGE to provide feedback regulation, however this is lost in TCDD-exposed UGSs.

Altogether, the *in vivo* findings demonstrate that around E16.5 in the ventral UGS, during normal development: (1) an as yet unidentified activating signal triggers the stabilization and nuclear localization of CTNNB1, (2) this nuclear CTNNB1 is functionally relevant since it upregulates expression of downstream target genes *Lef1* and *Wif1*, and (3) activation of AHR signaling in the UGS by TCDD can block the CTNNB1-stabilizing paracrine signal.

## 10. Wnt Signaling Components Upstream of CTNNB1 Altered by Activated AHR

Though we have demonstrated that *in utero* TCDD exposure inhibits canonical Wnt signaling in basal epithelial cells of the ventral UGS by preventing accumulation and nuclear localization of CTNNB1, the question remains, how? We performed a thorough examination of the Wnt signaling pathway by assessing the RNA abundance of over 40 genes that act upstream of CTNNB1. Pregnant C57BL/6J dams were treated with either vehicle or TCDD on E15.5, and UGSs were analyzed by ISH on E16.5 for differences in transcript abundance. The study revealed that RNA abundance of *Rspo2*, *Wnt10a*, *Wnt16*, *Ror2*, and as already discussed, *Wif1* was altered in TCDD-exposed UGSs (Table 2) [152].

### 10.1. Rspo2 and Rspo3

**Figure 4:**
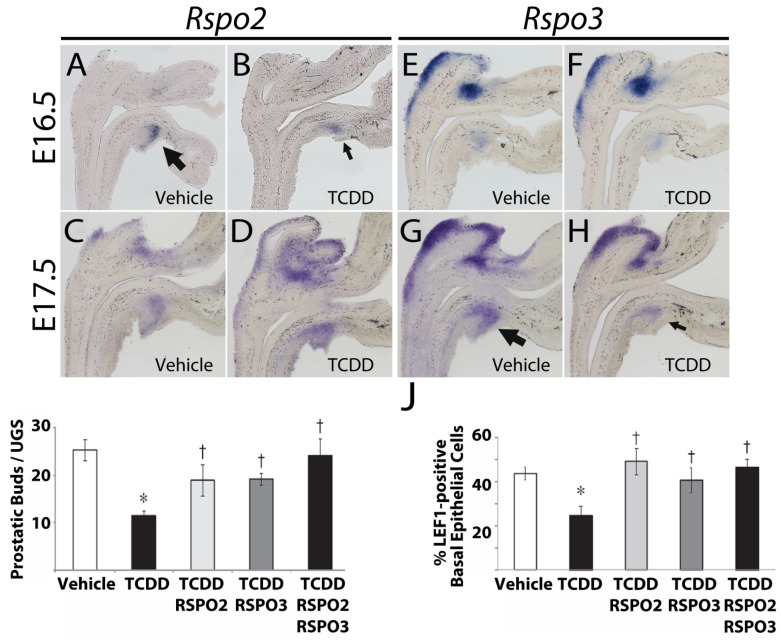
TCDD-induced activation of AHR signaling in the UGS of male, C57BL/6J mouse embryos downregulated *Rspo2* and *Rspo3* RNA abundance *in vivo*; UGS organ culture with exogenous RSPO2 and RSPO3 reversed inhibitory effects of TCDD-induced activation of AHR signaling *in vitro*. (**A**–**H**) Dams were dosed on E15.5 with corn oil vehicle (5 mL/kg, po) or TCDD (5 μg/kg, po). Images are representative sagittal sections from *in utero* vehicle-exposed (**A**, **C**, **E**, **G**) or TCDD-exposed (**B**, **D**, **F**, **H**) UGSs stained by ISH (purple stain). (**A**–**D**) *Rspo2* transcript abundance was reduced in TCDD-exposed UGSs (**B**) compared to control UGSs (**A**) at E16.5, but no difference was observed at E17.5 (**C**, **D**). (**E**–**H**) *Rspo3* transcript abundance was not different between control and TCDD-exposed UGSs at E16.5 (**E**, **F**), but was reduced in TCDD-exposed UGSs (**H**) compared to control UGSs (**G**) at E17.5. Arrows point to the ventral mesenchymal pad; small arrows represent decreased staining compared to big arrows. (**I**–**J**)) *In vitro* UGS organ culture with TCDD (1 nM) plus exogenous RSPO2 (100 ng/mL), RSPO3 (100 ng/mL), or a combination of RSPO2 + RSPO3 (100 ng/mL each) increased the number of prostatic buds (**I**), and the percentage of LEF1-positive basal epithelial cells (**J**) compared to culture with TCDD alone. Asterisks indicate a significant difference compared to vehicle-exposed UGSs; daggers indicate a significant difference compared to TCDD-exposed UGSs (*p* ≤ 0.05). (**A**, **B)** adapted from [152], and (**I**, **J**) adapted from [143] with permissions from Oxford University Press.

R-spondins (RSPOs) have been shown to activate the Wnt signaling pathway by multiple mechanisms, including synergism with Wnt ligands, activation of noncanonical Wnt signaling through heparin sulfate proteoglycan binding, competition with DKKs (extracellular antagonists of canonical Wnt signaling) for receptor binding, and activation of LGR receptors to enhance canonical Wnt signaling [165]. *Rspo2* and *Rspo3* RNA abundance was assessed in control and TCDD-exposed UGSs at E16.5, E17.5, and E18.5. RNA from both genes was present in the ventral mesenchymal pad (VMP) at all times tested [143,152]. The VMP is a subcompartment of the UGM that is thought to mediate mesenchymal/epithelial crosstalk during prostatic bud development [166]. In TCDD-exposed UGSs, *Rspo2* transcript abundance was reduced in the VMP at E16.5, but not E17.5 or E18.5, while *Rspo3* transcript abundance was unchanged in the VMP at E16.5, but decreased at E17.5 and E18.5 (Figure 4A–H) [143,152].

These findings were confirmed *in vitro*. Male UGSs were harvested at E14.5 and cultured with or without TCDD for 2, 3, or 4 days. *Rspo2* transcript abundance was reduced in TCDD-exposed UGSs after two days in culture (similar to E16.5), but was not different from control UGSs after three and four days in culture [143]. Conversely, *Rspo3* transcript abundance in TCDD-exposed UGSs was not different from control UGSs after two days in culture, but was reduced compared to control UGSs after three and four days in culture (similar to E17.5 and E18.5, respectively) [143]. Exogenous RSPO2 and/or RSPO3 were subsequently added to the culture media to determine if the canonical Wnt activators could increase prostatic budding in UGSs co-cultured with TCDD. RSPO2, RSPO3, and a combination of RSPO2 plus RSPO3 all increased the number of prostatic buds that developed compared to UGSs cultured with TCDD alone (Figure 4I) [143]. Furthermore, IHC revealed that RSPO2, RSPO3, and RSPO2 plus RSPO3 treatments all increased the percentage of basal epithelial cells expressing LEF1 and HNF1A (canonical Wnt target genes) compared to TCDD treatment alone (Figure 4J) [143].

These findings are significant for several reasons: (1) TCDD-induced activation of AHR downregulated expression of RSPO2 and RSPO3, two potentially key paracrine regulators of ventral prostate bud development; (2) this downregulation occurred in the UGM which is known to be the site of action for TCDD-induced, AHR-dependent inhibition of prostatic budding; and (3) addition of exogenous RSPO2 and RSPO3 to UGS organ cultures countered the effects of TCDD-induced activation of AHR signaling by upregulating canonical Wnt signaling and increasing prostatic budding. We have already established that TCDD-induced activation of AHR signaling can inhibit canonical Wnt signaling; these findings elucidating RSPO regulation and function upstream of CTNNB1 provide one possible mechanism by which this inhibition occurs.

### 10.2. Wnt10a and Wnt16

**Figure 5:**
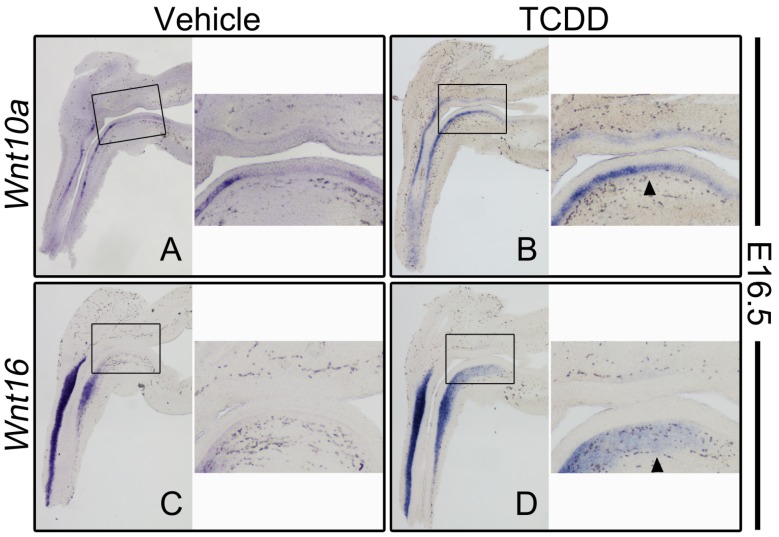
TCDD increases *Wnt10a* and *Wnt16* RNA abundance in the ventral UGS. Sagittal sections from *in vivo* vehicle-exposed (**A**,**C**) and TCDD-exposed (**B**,**D**) UGSs were stained by ISH (purple stain). (**A**) At E16.5 in control tissues, little to no *Wnt10a* RNA was detected in the ventral UGS. (**B**) *Wnt10a* RNA was detected in the ventral basal epithelial cells of TCDD-exposed UGSs. (**C**) At E16.5, little to no Wnt16 RNA was detected in the ventral region of control UGSs. (**D**) Wnt16 RNA was detected in the ventral mesenchyme of TCDD-exposed UGSs. Arrowheads point to *de novo* staining detected in the ventral UGS of TCDD-exposed UGSs. Reprinted from [152] with permission from Oxford University Press.

The ISH survey of Wnt signaling components acting upstream of CTNNB1 included all 19 of the known mouse Wnt ligands. TCDD exposure affected two Wnt genes, increasing RNA abundance of *Wnt10a* in the UGE, and *Wnt16* in the UGM [152]. More specifically, at E16.5 in control UGSs, *Wnt10a* transcript was observed in basal epithelial cells of the urethra, with little to no transcript present in basal epithelial cells of the ventral UGS (Figure 5A). In TCDD-exposed UGSs, *Wnt10a* staining was present not only in basal epithelial cells of the urethra, but also extended through the basal epithelium of the ventral UGS toward the base of the bladder (Figure 5B). Mirroring *Wnt10a* expression in the UGE, *Wnt16* RNA was observed only in the urethral mesenchyme in E16.5 control UGSs (Figure 5C), but detected in the urethral mesenchyme and ventral UGM in TCDD-exposed UGSs (Figure 5D). This is clear evidence of AHR signaling dysregulating the Wnt signaling pathway. However, it is not yet clear if activated AHR directly induced transcription of these genes, or if the increased RNA abundance is the result of AHR signaling affecting an intermediary that regulates *Wnt10a* and *Wnt16*.

*Wnt10a* is unlikely to play a significant role in inhibiting prostatic budding due to its expression in the UGE, however, *Wnt10a* is also expressed during palatal fusion [167], tooth development [168,169], skin, hair, and sebaceous gland development [170,171,172], and tail fin regeneration in zebrafish [115], all of which are disrupted by TCDD exposure [15,16,44,173,174,175]. Therefore, *Wnt10a* should be explored as a potential target in TCDD-induced toxicity in these tissues as well.

WNT16, on the other hand, is an attractive candidate as an inhibitor of prostatic budding. TCDD exposure induces WNT16 expression in ventral UGM, which directly interfaces with basal epithelial cells that give rise to ventral prostatic buds. In some systems, WNT16 has been shown to activate canonical Wnt signaling [176,177,178] but in others, WNT16 has no effect on CTNNB1 stabilization and instead, in some cases, activates non-canonical Wnt signaling [179,180,181,182,183]. The final outcome likely depends on what other agonists, antagonists, and receptors are present in the microenvironment [59,65,117,184]. We hypothesize that WNT16 antagonizes canonical Wnt signaling upstream of CTNNB1 in TCDD-exposed UGSs, perhaps in a manner similar to WNT5A in other systems [65,117,118,185,186,187]. Interestingly, WNT16, like WNT10A, is expressed during palatal fusion [167] and skin development [170,171,188] so *Wnt16* could be a target gene of AHR signaling in other tissues too.

### 10.3. Wnt5a

**Figure 6:**
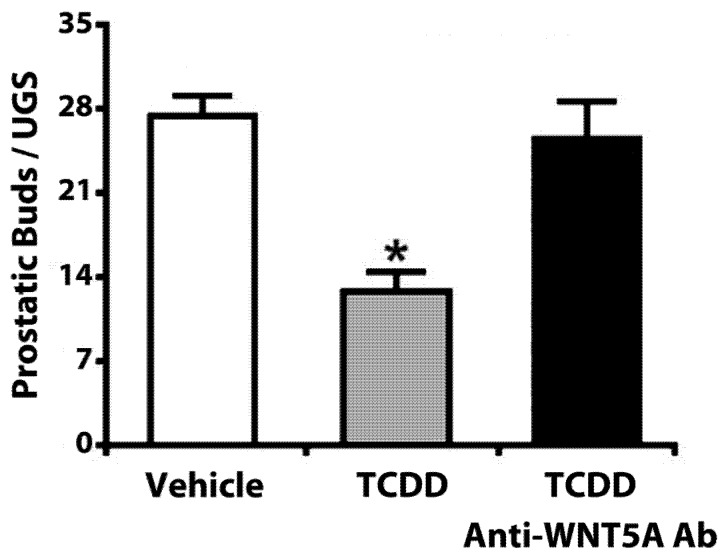
Anti-WNT5A antibody reversed the inhibitory effects of TCDD-induced activation of AHR signaling *in vitro*. UGSs from male C57BL/6J mouse embryos were harvested at E14.5 and cultured *in vitro* for three days. While culture with TCDD (1 nM) reduced total prostatic bud number, culture with TCDD (1 nM) plus a neutralizing anti-WNT5A antibody restored prostatic bud number to a level similar to that of control UGSs. An asterisk indicates a significant difference from control UGSs (*p* ≤ 0.05). Adapted from [153].

WNT5A is required for normal UGS and prostate development, and is expressed in the UGM during prostatic budding initiation and elongation, and during prostatic ductal branching [153,189]. Although exposure to TCDD did not alter WNT5A expression in the UGS [152,153], we found that adding a neutralizing anti-WNT5A antibody to UGSs cultured with TCDD restored total bud number to a level similar to that of control UGSs cultured without TCDD (Figure 6) [153]. Furthermore, culture with exogenous WNT5A decreased total bud number and impaired ductal outgrowth and branching [153,189]. Therefore, while some WNT5A activity is required, in general, it appears that WNT5A is antagonizing prostate development.

## 11. Intersection of AHR and Wnt Signaling in the UGS

**Figure 7:**
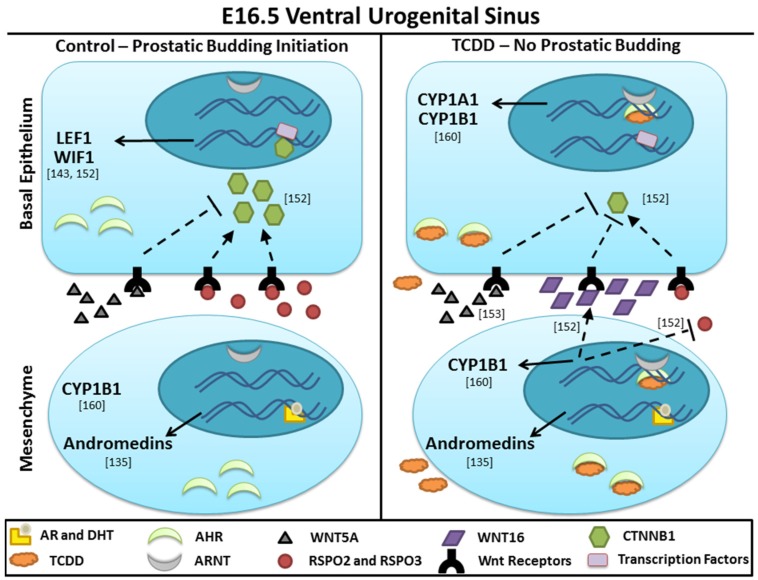
Hypothetical mechanism by which TCDD-induced activation of AHR signaling in the ventral UGM inhibits prostatic budding in the ventral UGE. Andromedins are paracrine factors induced by androgen signaling and expressed in the UGM that promote prostatic budding initiation. The identity of andromedins is not fully known. WNT5A, RSPO2, and RSPO3 are not the only ligands present in the extracellular matrix that are capable of activating Wnt signaling, but the others are not shown. The Wnt receptors on the epithelial cell surface have not been defined because it is not yet known which receptor or combination of receptors is present. The transcription factors in the epithelial cells include members the TCF/LEF family. CYP1B1 is expressed in the UGM during normal development, but it is not known what induces its expression. CYP1B1 expression was increased in the UGM after TCDD exposure. Arrows represent upregulation; blunt-end lines represent downregulation. Solid lines indicate a known outcome; dashed lines indicate some degree of uncertainty and further research is required. For example, it is known that activation of AHR in the UGM upregulates *Wnt16* in the UGM and downregulates *Rspo2* and *Rspo3* in the ventral mesenchymal pad, but it is not known if these are direct or indirect effects of activated AHR. References are in brackets. We hypothesize that to initiate prostatic budding there are multiple Wnt signaling events that, on balance, generate a precisely regulated activation of the canonical Wnt signaling cascade. In TCDD-exposed UGSs, this balance is disrupted resulting in either failed or blocked activation of canonical Wnt signaling.

Our current hypothesis of the paracrine mechanism by which *in utero* TCDD exposure inhibits ventral prostatic budding in male mouse embryos—activation of AHR signaling in UGM alters Wnt signaling in UGE—is illustrated in Figure 7. The genes shown in Figure 7 are almost certainly not the only Wnt signaling regulators that play a role in prostatic bud formation; instead, they are the genes we have found to be dysregulated by TCDD-induced activation of AHR signaling.

Multiple studies have shown that CTNNB1 signaling is required in the UGS for prostatic budding [66,121,144]. We have extended these findings by showing that, just prior to budding initiation, under normal developmental conditions (Figure 7, left panel), CTNNB1 accumulates in basal epithelial cells, translocates to the nucleus, and activates transcription of downstream target genes (*Lef1*, *Wif1*) [152]. The upstream signal(s) that induce CTNNB1 accumulation are not yet known, but could involve RSPO2 and RSPO3 [143]. WNT5A is also present in the UGM during this developmental window and could be dampening CTNNB1 signaling to prevent over-activation, which can also hinder budding. In TCDD-exposed UGSs (Figure 7, right panel), the paracrine signals originating in the ventral mesenchyme are antithetical to prostatic bud development. Activation of AHR signaling in the UGM promotes WNT16 expression in the UGM, though the mechanism by which this occurs has not yet been elucidated [152]. We hypothesize that WNT16 synergizes with WNT5A to enhance an inhibitory signal that ultimately prevents activation of CTNNB1 signaling by inhibiting CTNNB1 accumulation and nuclear localization. In culture, a neutralizing anti-WNT5A antibody reduced this inhibitory signal, causing increased CTNNB1 signaling in the UGE that resulted in increased budding [153].

Interestingly, during normal development, both WNT5A and WNT16 are expressed in the mesenchyme around the urethra where no buds develop, while only WNT5A is expressed in the ventral UGM. After exposure to TCDD both WNT5A and WNT16 are expressed in the ventral UGM and no prostatic buds develop [152].

## 12. Conclusions

Both AHR and Wnt signaling have been rigorously studied for many years, but not until relatively recently was there evidence of crosstalk between these two pathways. Our review of literature exploring the intersection of AHR and Wnt signaling in vertebrates found a majority of these studies reported that activated AHR downregulated canonical Wnt signaling (Table 1 and Table 2). Carefully regulated Wnt signaling is essential for normal development and adult tissue maintenance. Both insufficient and excessive signaling can be deleterious. It seems reasonable to hypothesize that an innate function of activated AHR is to fine-tune Wnt signaling, keeping it within a biologically “acceptable” level. Importantly, this crosstalk might be a useful tool in the treatment of disease. For example, many cancers have excessive canonical Wnt signaling. Pharmaceutical manipulation of AHR activity could prove to be one prong of a multi-pronged, personalized treatment plan for such cancers.
